# The Unite for Diabetes campaign: Overcoming constraints to find a global policy solution

**DOI:** 10.1186/1744-8603-4-3

**Published:** 2008-02-19

**Authors:** Karen Siegel, KM Venkat Narayan

**Affiliations:** 1 Associate, MATRIX Public Health Solutions, Inc., 85 Willow Street Suite 3, New Haven, CT, 06511, USA; 2 Hubert Professor of Global Health and Epidemiology, Hubert Department of Global Health, Rollins School of Public Health, Emory University, 1518 Clifton Road NE Atlanta, Georgia, 30322, USA

## Abstract

Despite the fact that diabetes and other non-communicable diseases represent a significant proportion of the global burden of disease, proportionate global action has not occurred. A 2003 article reported on global constraints to the implementation of effective policies to curb non-communicable disease epidemics. These constraints include a lack of global advocacy, insufficient attention from funding agencies and governments, partnerships and interactions, capacity and resources, and global norms and standards, as well as orientation of health services to acute care. Building on these ideas, this paper will review the progress that has been made with regards to each constraint, focusing on the International Diabetes Federation's Unite for Diabetes campaign and United Nations resolution on diabetes to show how this event – driven by globalization – has helped remove some of these barriers. Additional progress in diabetes and NCD prevention and control is also highlighted. The paper concludes by outlining what still needs to happen for globalization to be an effective solution for diabetes and non-communicable disease prevention and control.

## Introduction

### A Global Problem

Every 10 seconds, someone in the world dies of diabetes-related causes, placing the gravity of the diabetes epidemic at least on par with that of HIV/AIDs. In the same 10 seconds, another two people are diagnosed with the disease for the rest of their life [1] and may suffer increased morbidity and reduced quality of life, premature death, and large adverse economic effects due to higher healthcare and non-healthcare costs. Diabetes causes an estimated 12–14 years of life lost to premature death. [2] In the United States in 2002 people with diabetes spent six times more money on healthcare than people without diabetes; mostly due to costly complications like cardiovascular disease, renal failure, and blindness. Morbidity, mortality, and quality of life for people living with diabetes in low- or middle-income countries may decline further if insulin and appropriate health care is neither available nor accessible. Currently, 246 million people have diabetes, and if no action is taken, this number will increase to over 380 million in the next 20 years. Approximately 5–10% of all people with diabetes have type 1, while the remaining 90–95% accounts for type 2 diabetes, which is largely related to lifestyle and responsible for most of the increase. An additional 200 million people around the world have impaired glucose tolerance, a precursor for type 2 diabetes, and this figure is expected to rise to 420 million by 2025. [3]

Once considered a "disease of affluence," diabetes now places a significant burden on developing countries. Globally, diabetes affects 5.9% of the adult population, but in many countries in Asia and the Pacific, diabetes affects up to one-third of the population. 80% of the global diabetes burden is in the developing world, and emerging economies are particularly susceptible, since undernourishment and stunting in childhood often leads to later onset of diabetes; this, combined with rapidly changing environments due to the nutrition transition and urbanization, puts future working-age populations at high risk. [4] China and India alone make up 25% of the total diabetes burden, and face large increases in the next two decades, with 104% and 150% escalations, respectively. This will have profound consequences on the two rapidly developing populous economies.

### Global Consequences

Diabetes is of significance because of the social, economic and health burden it places on countries, and on individuals and their families. Costs of diabetes are manifested in both direct and indirect costs that put pressure on individuals, societies and governments. Direct costs include medical costs for long-term care and complications; indirect costs account for losses in productivity, coping mechanisms, and the costs of quality of life, which affects individuals and families and is immeasurable. In many countries, the cost of insulin and diabetes supplies far exceeds annual incomes, leaving people with diabetes unable to properly manage their condition, and susceptible to complications in the long-term. Treatment of complications is more expensive than prevention or control, and studies have shown that health care expenditure for people with diabetes is five times higher than for people without diabetes. [5] In low income populations in urban India, annual income spent on diabetes-related health care increased from 24.5% in 1998 to 34.0% in 2005, 93.6% of which is out-of-pocket. [6]

As the diabetes epidemic unfolds, individuals are being affected at younger ages due to increased risk exposure. Globally, type 2 diabetes disproportionately affects the working age population; 46% of those affected are aged 40–59 years, and half of all diabetes-related deaths occur in individuals under age 70. For families, diabetes can be a death sentence and a straight path to poverty if the person with diabetes is the sole breadwinner – disabilities from diabetes complications can lead to productivity loss and life-long care. The WHO estimates that China, the Russian Federation and India will lose $558 billion, $303 billion, and $237 billion, respectively, in foregone national income due to diabetes, stroke and heart disease in the next decade. [7]

Despite these facts, diabetes – and most non-communicable diseases (NCDs) – has been largely neglected, due to lack of financial and human capital, lack of fully-informed key decision makers, and orientation of health systems toward acute care. [8] Donors tend to fund issues that are more easily addressed, such as vaccines and treatment of acute diseases, and want to see rapid results. In developing countries where infectious diseases persist, chronic diseases are viewed as secondary in importance. Lack of up-to-date information and education are crucial factors in gaining support for NCD prevention and control. Misconceptions still exist; NCDs are due to lifestyle choices and should be the responsibility of the individual, NCDs only affect the rich and the elderly, infectious disease rates far outweigh NCD rates – as illustrated above, these beliefs are false. Furthermore, elected officials tend to react to problems that have immediate solutions and can produce results within their term. Diabetes by nature requires long-term investment; prevention and control efforts implemented today will only produce visible results in one decade. The media also shapes how health issues are framed and addressed: children dying of HIV/AIDs or diarrheal diseases provide a more compelling image than individuals affected by diabetes. However, there need not be such polarization of the issues: NCD prevention efforts have much to learn from infectious disease successes, and vice-a-versa.

### Globalization as a Driver

Increasing diabetes rates are driven by factors outside of the health sector. Globalization refers to the 'process of increasing the connectivity and interdependence of the world's markets and businesses', [9] as well as the increasing movement of ideas, people, commerce and financial capital. [10] This leads to changes in cultural norms, lifestyle, food supply, and ideas. Although not a new phenomenon, globalization's scale and pace are rapidly increasing, due to improvements in IT and transportation, and due to greater integration of supply chains.

Globalization facilitates the spread of three risk factors (poor diet, physical inactivity, tobacco use) that lead to four diseases (cardiovascular disease, some cancers, chronic respiratory disease and diabetes) which cause more than 50% of deaths worldwide. In 2001, diabetes accounted for 19,996,000 disability-adjusted life years, 80% of which were in developing countries, especially in East Asia and the Pacific. This represented a 250% increase worldwide from 1990, and a 266% increase in low and middle income countries. [2,11]

These risks are attributable to the nutrition transition, accelerating technological advances that bring time-saving gadgets and rapid methods of transportation that discourage physical activity, changes in workplaces and work hours, increasingly aggressive global marketing campaigns and urbanization. The nutrition transition refers to the replacement of traditional diets high in fruits and vegetables with a diet high in calories, animal fat and vegetable oils, and processed foods, and is occurring in most developing countries throughout the world. [12] Global economic policies concerning agriculture, trade, investment and marketing affect what the world eats, as do global food and health policies. [13] Trade liberalization has led to increased supply, decreased prices and increased marketing. [14] According to JP Morgan, globally "the cost of a calorie (energy) has fallen dramatically over the past couple of years on the back of fat products falling in price by more than 50% over the past 50 years (carbohydrates to a lesser extent) while vegetables increased by more than a third." [15] Additionally, increased access to and affordability of energy-dense foods, relative to nutrient rich foods, leads to consumption of these unhealthier foods. Resulting high-calorie diets rich in fat and low in fruits and vegetables, coupled with reduced energy expenditure in urban environments especially and marketing campaigns encouraging overconsumption portend obesity, which leads to non-communicable diseases (NCDs), including type 2 diabetes.

Although globalizing forces can result in adverse health outcomes, health is vital to the future of globalization. In almost every country throughout the world, health produces wealth, and wealth produces health. [16,17] A failure to invest now in health will be disastrous for countries when today's children become the next generation of workers. As emerging market countries integrate into the global economy, the health and vitality of their workforce is imperative. According to Steve Leeder and colleagues, "Just at the time when developing countries' economies have the opportunity to invest more of their capacity because a brief window of lower dependency has opened, the workforce that nations count on to exploit that opportunity is itself prematurely dying". [18] Such global problems require global and local solutions.

## Global progress in diabetes prevention and control

### Overcoming global constraints

A 2003 article outlined five constraints to effective global policies to curb increasing NCD rates: a lack of ***global advocacy, partnerships and interactions, capacity and resources, global norms and standards***, as well as a ***health service orientation ***towards acute care. [19] The authors noted that these constraints give powerful opposition to policies and interventions for promotion of healthy eating and physical activity, and concluded that until the barriers are overcome, progress will be slow for NCD prevention and control. Table 1 summarizes these ideas.

**Table 1:** Global Responses and Progress in 2003

**Global Response**	**Progress in 2003**	**Example**
Global advocacy	"What there is tends to be fragmented and risk-factor or disease specific"	
Partnerships and interactions	"If widely implemented, changes [especially in food industry] could harness the benefits of globalization and promote health"	WHO Strategy for Diet and Physical Activity, some progress with food (Kraft) and alcohol industry
Capacity and resources	"national capacity for non-communicable disease prevention and control is weak and the institutional response to capacity development has not kept pace with epidemiological transition"	NIH and Fogarty International Center
Global norms and standards	"increasing need to establish global norms...treaties are not the solution to the complex issues related to nutrition transition or physical inactivity. Multistakeholder and intergovernmental mechanisms and other non-binding measures are better options, especially in relation to children"	FCTC
Reorientation of health services	Prevention, treatment and palliative care not implemented in most countries; focus on acute care	

Indeed, progress has been slow in gaining proportionate responses to the diabetes pandemic. However, the recent, ongoing Unite for Diabetes campaign, combined with other global developments, mark progress in overcoming these constraints and suggests future success in NCD prevention and control. The campaign has united the global diabetes community, and led to the passage of a UN Resolution on Diabetes (UNR) in December 2006. The following section describes the campaign, illuminating progress that has been made in terms of the five constraints to global policies as a direct result (Table 2), and highlights areas that still need to be addressed for globalization to effectively prevent and control NCDs.

**Table 2:** Global Responses and Progress resulting from the Unite for Diabetes campaign

**Global Response**	**What the Unite for Diabetes campaign adds**	**Example/Achievement**
Global advocacy	Global coalition of 190 IDF member associations from 150+ countries Campaign kits provided to members of the global diabetes community Blue circle and pins for solidarity Includes youth	Changing Diabetes Barometer allows for measurability of the diabetes pandemic, crucial for driving global awareness and action
Partnerships and interactions	Collaboration between IDF and pharmaceutical companies [Novo Nordisk, GlaxoSmithKline, LifeScan, Lilly, Merck, Pfizer, Bayer HealthCare, Lloyds Pharmacy, Novartis, Sanofi Aventis, Abbott Diabetes Care] Largest ever diabetes coalition of IDF member associations, professional societies, charities and industry	Novo Nordisk Changing Diabetes Leadership Forum brought together policymakers, government officials, international and patient organizations, healthcare professionals, people with diabetes and media from 20 countries to address diabetes needs
Capacity and resources	Gives countries capacity and encouragement to develop national diabetes plans; resources should follow as countries place diabetes higher on agendas Motivates global diabetes community to coordinate better	World Diabetes Foundation The December 2006 passage of the UN Resolution will facilitate these processes.
Global norms and standards	UNR urges all governments to create national diabetes plans for the prevention, treatment and care of diabetes	
Reorientation of health services	UNR calls on all nations to develop national policies for the prevention, treatment and care of diabetes	

### Unite for Diabetes campaign

#### The campaign

The Unite for Diabetes campaign and UNR was the brainchild of 20 year old Clare Rosenfeld and her mother Kari Rosenfeld, who realized three years ago that the diabetes world needed more cohesion to ensure recognition and adequate treatment and care for all people with diabetes. Officially launched in June 2006, the IDF-led Unite for Diabetes campaign aims to:

• Place diabetes on the global agenda

• Increase awareness of the disease and patient education

• Address poverty as a main obstacle to access to quality healthcare and insulin

• Pass a UN Resolution on Diabetes, which calls on all governments to create national plan for the prevention, treatment and cure of diabetes.

The campaign has two main parts – a top-down approach targeting major policymakers around the world, and a bottom-up approach aiming to make 1 billion people aware of diabetes and the campaign. According to C.K. Prahalad, incorporating the 4 billion people living in poverty (less than $2 per day) in profitable win-win engagements can help to alleviate poverty and associated problems, in this case access to healthcare and essential diabetes medicines. One overarching goal is to become more efficient – and thus more effective – as a group [20] and to overcome barriers to prioritizing diabetes on a global scale. The Unite for Diabetes symbol, a blue circle representing unity and the color of the sky which unites us all, is used to spread awareness of diabetes, similar to the way in which red ribbons promote AIDS awareness and recognition as a public health priority.

#### Global Advocacy

To address the bottom-up part of the campaign, a global coalition of diabetes associations is engaged, as well as two groups of youth: the Novo Nordisk Youth Panel (NNYP) and IDF Youth Ambassadors.

NNYP is a group of nineteen young people – fourteen of whom have diabetes – from twelve countries. The Panel's contribution to the campaign is to spread awareness amongst the targeted 1 billion people by creating National Youth Panels of diabetes advocates in their respective home countries, and through the internet. Panelists have employed information campaigns on World Diabetes Day, blogs that highlight the issues, press release, newspaper articles and radio/television interviews, lobbying and peer-to-peer communication to build support among the public and among politicians. To date, a National Youth Panel and website has been set up in Spain, El Salvador hosted a diabetes fair that was attended by Health Minister Dr. Guillermo Maza Brisuelas, who pledged his support of the UNR, and in Italy one panelist has spoken with important policymakers in the EU, including Franco Frattini, the current vice-president of the European Commission.

The IDF Ambassadors, 25 young people with diabetes from developing and developed countries, met in Cape Town, South Africa at the 2006 IDF conference to participate in global diabetes advocacy. Sponsored by Novo Nordisk and the IDF, the Youth Ambassadors collaborated with each other on how to best engage in diabetes issues in their own countries as youth ambassadors, presented their conclusions at a conference session, engaged with professionals at the conference, and created a video, "Break the Silence," which illuminates challenges faced by people with diabetes around the world. At the end of the conference, the youth returned to their home countries to tackle diabetes on a national level, spreading awareness among policymakers and the general public, ensuring the UNR's implementation in their own country based on country-specific priorities.

Further advocacy was created, in part through these two groups, on November 14, 2007, the first official World Diabetes Day. The worldwide event was a celebration of the campaign's success, and involved advocacy events at the United Nations building in New York and other global landmarks.

#### Partnerships and interactions

The campaign brought together the largest ever diabetes coalition (190 IDF member associations, scientific and professional diabetes societies, charitable foundations and service organizations, industry and youth) and benefited from a partnership between Novo Nordisk and the IDF. Engagement and unity of so many diverse actors from different backgrounds has the potential to solve practical public health problems, as each brings unique talents, resources, and perspectives to the table.

In September 2006, Novo Nordisk launched a Changing Diabetes Bus, a global drive for change. The goal is to raise awareness of diabetes and its social, humanitarian and economic consequences on a one-and-a-half-year long journey around five continents. The hope is to communicate to important stakeholders the urgency of the diabetes pandemic. [21] Novo Nordisk's involvement in the campaign also stimulated competitive spirit in other pharmaceutical companies such as Merck, who then joined in as well with resources for geocaching activities to foster campaign awareness.

However, since most of the drivers of diabetes lay outside of the health sector, future partnerships should strive to go beyond pharmaceutical and medical groups. Partnerships with industry can help to harness the benefits of globalization and public health promotion, and should be cultivated. Recent successes include a recent partnership between the Department of Health in the UK, Jamie Oliver and Sainsbury's, which harnesses Sainsbury's corporate strength and Oliver's immense popularity among children to suggest what parents and children to promote healthy family meals as a way of improving the health of the nation. [22] In May 2006, collaboration between the Alliance for a Healthier Generation (a partnership between the William J. Clinton Foundation and the American Heart Association), the American Beverage Association and the three largest soda companies in the world – Coca-Cola, PepsiCo and Cadbury Schweppes led to an agreement to ban soda sales in elementary and middle schools in the United States, which was implemented in late 2006. Moreover, Nike and Apple's unique collaboration in the Nike+ system harnesses market innovation to inspire physical activity among consumers. [23] With particular respect to a lack of availability and access to insulin in many developing countries, collaboration and partnerships with pharmaceutical companies that can help deliver essential medicines and health education to the people who need it are crucial.

#### Capacity and resources, Global norms and standards

In many developing countries, national capacity is absent or weak due to a strong focus on infectious diseases – national capacity can be strengthened by global action, as highlighted in a recent Nature article. [24]

On December 20, 2006, the United Nations General Assembly passed the landmark resolution, co-sponsored by Bangladesh and South Africa, the current leader of the G77. In doing so, the UN recognized the global threat of the diabetes epidemic and placed a NCD on their global health agenda for the first time. The Resolution names the current IDF World Diabetes Day, November 14th as a United Nations Day to be observed every year starting in 2007, and calls on all nations to develop national policies for the prevention, treatment and care of diabetes in line with sustainable development of their healthcare systems. The Resolution does not mobilize funds, but urges national governments to allocate resources for diabetes action.

With implementation, the UNR strengthens countries with less responsive governments, and gives legitimacy to national effects that might be undermined without a global backdrop. The Framework Convention on Tobacco Control (FCTC) and Global Strategy for Diet and Physical Activity, which pulled together players from all over the world, formed similar alliances that were met with success. According to Yach et al., the FCTC created a global forum to highlight issues, promotion of multilateral coordination and domestic action, facilitation of the development of national coalitions, and the mobilization of NGOs, media and the general public. [25] The FCTC led to UN system-wide approach to tobacco control and national, complementary actions which would not have been possible in the absence of a global alliance and the synergistic effect of players from multiple sectors working together. The passage of the UNR follows in the footsteps of the FCTC; it has helped to develop capacity, and national, complementary actions and resources will hopefully follow as diabetes is recognized as higher priority. It will also lead to global norms and standards as all countries begin to develop harmonizing diabetes prioritization strategies. Still, no accepted list of essential drugs and diagnostics for diabetes and other NCDs exists, suggesting a critical area of neglect that needs to be addressed, since high costs result from diagnosis as well as treatment, but are rarely tackled together.

#### Health service reorientation

The UNR calls on all nations to develop national policies for the prevention, treatment and care of diabetes in line with sustainable development of their healthcare systems. A key message is that diabetes care should be defined as a human right. Insulin (as well as equipment and education) is a basic tool for survival for people with diabetes and is crucial to successful diabetes control strategies that can prevent costly complications down the road. Additionally, patient education is cost saving, and is paramount to successful management and control of diabetes. [26] Primary and secondary care centers should be accessible and affordable, and national diabetes screening guidelines should be implemented to ensure early detection and diagnosis and ongoing care. Monitoring of governments (surveillance) is needed to a.) make sure governments have a plan and follow through with it and b.) allocate appropriate funds. Lastly, countries should strive to increase human resources for prevention and control of diabetes; India's Health Minister recently created an effort to provide incentives and encourage Indian physicians and public health professionals living abroad to return to India; a recent review of global public health schools shows a considerable a lack of NCD capacity-building and research in developed and developing countries – an enormous barrier to improved prevention, control and management. [27]

#### Future progress

Much progress has been made, but much more remains to be done. A key aspect of the campaign is that it is disease-specific. The benefit is that it focuses attention directly on diabetes, a condition that has historically received little recognition, even compared to other NCDs like CVD. However, future steps should closely align diabetes efforts with other NCD and obesity efforts for maximum benefit and unity. Even type 1 diabetes, which is not yet preventable, is linked to CVD, blindness and chronic renal failure; advocates can benefit from global alliances with advocates of conditions that share common risk factors and complications. Governments should be encouraged to address all lifestyle related chronic diseases: cardiovascular diseases, cancers, respiratory diseases. Structural emphasis on research and development, low cost sustainable interventions, and policy analysis should all be incorporated into these efforts. Global norms regarding marketing to children, labeling of foods, and industry incentives for producing acceptable fruit and vegetable-based products at low cost could also help to encourage healthier lifestyles and prevent diabetes.

More players – the WTO and others – should be included in discussions as countries develop national diabetes policies, ensuring that trade and other policies complement national health policies. Specifically, more industry players should be included in efforts. The private sector, especially food, sports, and retail industries are becoming increasingly involved in obesity prevention and control, which goes hand in hand with diabetes prevention and control. Industry can – and should – engage in spreading awareness of diabetes and other NCDs; many have already begun. [28] There is an opportunity for the trademark blue circle to be placed on packaging or other industry-materials, aligning companies with the cause for diabetes, and further spreading awareness of the Unite for Diabetes campaign – a win-win partnership. A broader alliance is more likely to generate the political support needed to make real progress in diabetes prevention and control.

#### Missed Opportunity?

Despite the campaign's success, lessons can be learned for future improvements. A prolonged Unite for Diabetes campaign could have produced much more media attention, public awareness, and policymaker support than it did. Launched in June 2006, the campaign gained momentum, but ended abruptly six months later with the UNR's passing. Diabetes associations and groups around the world reported on the campaign's success, but preaching to the choir will not raise awareness among the general public of diabetes as a global health priority. Media attention in news sources around the world was absent, with no major newspaper reporting on the passage of the UNR. Incorporating multi-stakeholder involvement and activism in the passage of the UNR could have gained more buy-in, and potential for political support.

Certainly policy is what will create action by governments around the world, but perhaps this could be facilitated by increased awareness? Strengthening the bottom-up approach can make policy change easier. Intractable challenges include a lack of political will, misconceptions about the causes and consequences of diabetes, and lack of funding for turning research into action and public health practice, all of which could have been addressed with a longer campaign.

## Conclusion

Past literature has focused on globalization and spread of unhealthy lifestyles as a negative player in global health. Globalizing forces can also facilitate the spread of best practice for diabetes prevention and control, and help to overcome the five global constraints discussed in this paper.

Harnessing the power of globalization has the potential to create real change for diabetes communities throughout the world, as countries with less capabilities benefit from strong global coalitions. The Unite for Diabetes campaign and subsequent passage of the UNR, as well as other recent global initiatives, provides a clear example of globalization's positive effect on health in the fight to place diabetes higher on global health and political agendas, and can be used as a model for future endeavors.

In addition to the Unite for Diabetes campaign and UNR, much progress has recently been made for NCD prevention and control, with more players stepping up to the plate since 2006. Strong partnerships, resulting in increased capacity and resources, have been formed, such as the Ovations Chronic Disease Initiative and the 2007 launch of Community Interventions for Health, a multinational community-based program to reduce the risk factors for chronic diseases in China, India, Mexico and the UK. [29,30] In November 2007, the Oxford Health Alliance's Grand Challenges initiative's Phase 1 was completed, which has identified 20 global policy and research priorities for addressing NCDs and formed the Grand Challenges Global Partnership. [24] Many countries are beginning to shift health systems to be more prevention-focused; in the UK, for example, the government is taking aggression action to address obesity and NCDs, and in January 2008 the Indian Health Minister announced the launch of a pilot National Diabetes Program.

These events, coupled with the Unite for Diabetes campaign and passage of the UNR mark an important turning point in viewing diabetes and other chronic diseases as global health priorities, and hopefully will result in more global initiatives, sustained funding increases, media and policy attention, and to change attitudes and behaviors. Although the UNR is only a piece of paper, its implementation will hopefully lead to real action that will also have real impacts.

## Competing interests

KS is a member of the Novo Nordisk International Youth Panel.

## Authors' contributions

KS drafted original the manuscript. KMVN provided expert revisions, helpful comments and suggestions, and reviewed the manuscript for intellectual content. Both authors read and approved the final manuscript.
